# Metabolic Signature of Arsenic Exposure and Metabolism: The Folic Acid and Creatine Trial

**DOI:** 10.1021/acs.est.5c01597

**Published:** 2025-07-16

**Authors:** Wending Li, Haotian Wu, Jeff Goldsmith, Ronald A. Glabonjat, Vesna Ilievski, Olgica Balac, Vesna Slavkovich, Brismar Pinto-Pacheco, Xiangping Lin, Faruque Parvez, Gabriela L. Jackson, Abu B. Siddique, Mohammad Nasir Uddin, Tariqul Islam, Irene Martinez-Morata, Ana Navas-Acien, Megan M. Niedzwiecki, Marianthi-Anna Kioumourtzoglou, Brandon L. Pierce, Joseph H. Graziano, Teodoro Bottiglieri, Douglas I. Walker, Mary V. Gamble

**Affiliations:** † Department of Environmental Health Sciences, Mailman School of Public Health, 5798 Columbia University, New York, New York 10032, United States; ‡ Department of Biostatistics, Mailman School of Public Health, Columbia University, New York, New York 10032, United States; § Department of Environmental Medicine and Climate Science, 5925 Icahn School of Medicine at Mount Sinai, New York, New York 10029, United States; ∥ Department of Genetics, Stanford University School of Medicine, Stanford, California 94305, United States; ⊥ Columbia University Arsenic Project in Bangladesh, Dhaka 1450, Bangladesh; # Department of Public Health Sciences, 2462 University of Chicago, Chicago, Illinois 60637, United States; ∇ Department of Human Genetics, University of Chicago, Chicago, Illinois 60637, United States; ○ Comprehensive Cancer Center, University of Chicago, Chicago, Illinois 60637, United States; ◆ Center of Metabolomics, Institute of Metabolic Disease, Baylor Scott and White Research Institute, Dallas, Texas 75226, United States; ¶ Gangarosa Department of Environmental Health, Rollins School of Public Health, Emory University, Atlanta, Georgia 30322, United States

**Keywords:** arsenic, metabolomics, folic acid, one-carbon metabolism, randomized controlled trial

## Abstract

Arsenic exposure remains a leading public health concern. Folic acid (FA) supplementation enhances one-carbon metabolism (OCM), thus promoting arsenic methylation and facilitating urinary arsenic elimination. Here, we investigate the metabolic profiles linked to arsenic exposure and metabolism based on a FA clinical trial. Arsenic exposure was assessed by the concentrations of blood arsenic (bAs) species: arsenite [InAs^III^], arsenate [InAs^V^], monomethyl- [MMA], and dimethyl- [DMA] arsenicals. Arsenic metabolism was assessed by the relative distribution (%) of these arsenicals in urine. Nontargeted metabolomic profiling was analyzed by LC-HRMS. OCM-related metabolites were analyzed by HPLC/MS/MS. Metabolomic profiling identified 8 unique metabolites and 812 metabolomic features (FDR < 0.05) associated with bAs (predominantly As^V^), and 66 metabolites and 285 metabolomic features with %uAs (predominantly %uInAs). Metabolic pathways enriched for bAs and %uAs were similar, highlighting phenylalanine, tyrosine, and tryptophan biosynthesis. A FA-induced %uInAs change was associated with four metabolites, three of which share links to acetyl-CoA metabolism. Of 11 measured OCM metabolites, cystathionine was positively associated with all bAs species. Methionine, S-adenosylmethionine, S-adenosylhomocysteine, cysteine, choline, betaine, and dimethylglycine were associated with increased As methylation profiles in urine (FDR < 0.05). Collectively, these findings may aid in the discovery of mechanisms underlying arsenic-induced health outcomes and potential targeted interventions. This trial was registered with clinicaltrials.gov: NCT01050556

## Graphical abstract

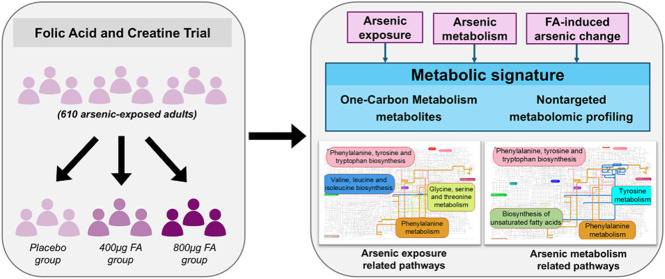

## Introduction

Over 140 million people worldwide are chronically exposed to arsenic-contaminated drinking water at concentrations above the WHO provisional guideline of 10 μg/L.^1,2^ Chronic arsenic exposure is associated with increased risk for diseases including cardiovascular disease,^3^ metabolic syndrome,^4^ neurologic impairments,^5^ and cancer.^6–8^ Reducing and mitigating the impact of chronic arsenic exposure therefore is a global public health priority.

Arsenic in drinking water is inorganic (InAs). Once ingested, InAs is metabolized by arsenic-3-methyltransferase (AS3MT), which transfers methyl groups from S-adenosylmethionine (SAM) to InAs to form monomethyl (MMA) and dimethyl (DMA) arsenicals. This process is dependent on one-carbon metabolism (OCM) for the synthesis of SAM and facilitates urinary arsenic elimination. Studies have shown that differential arsenic metabolism capacity, as indicated by the relative proportion of total urinary arsenic as InAs (%uInAs), MMA (%uMMA), and DMA (%uDMA) can affect future disease risk.^4,9–11^ Thus, measures targeting OCM may provide new insights to tackling the public health crisis of arsenic contamination globally. The Folic Acid and Creatine Trial (FACT) is a unique study designed to investigate the effect of folic acid (FA) and creatine, two key players in OCM, in increasing arsenic elimination in an arsenic-exposed Bangladeshi population. Our prior findings revealed that FA supplementation can enhance arsenic methylation^12^ and thereby increase urinary arsenic excretion and reduce blood arsenic levels.^13–15^ However, the potentially pleiotropic downstream effects of increasing arsenic metabolism through FA supplementation remains to be explored.

Several studies have investigated the metabolome in relation to arsenic exposure in humans.^16^ Most of these studies were conducted in populations with chronic health conditions^17–21^ or mothers and newborns,^22–24^ which may have limited generalizability.^25,26^ In general populations, evidence is scarce and exists only for selected arsenic markers and matrices.^27–29^ These studies were limited in sample size, focused on total and/or inorganic arsenic in urine or drinking water, and employed targeted approaches. Furthermore, available studies assessed urinary metabolome, which can be influenced by hydration status and renal function, and may not fully capture the systemic metabolomic changes underlying arsenic metabolism and toxicity.

Recently, we conducted a pilot study of blood metabolomic profile of 60 FACT participants.^30^ Our preliminary analysis identified 17 metabolites associated with blood arsenic species, with arsenate (As^V^) and DMA showing distinct association patterns, as expected given the differential toxicity of these species. However, owing to the small sample size and the pilot design, that study primarily focused on blood arsenic species, assessed a limited number of metabolites and features, and did not assess the effect of FA intervention-induced arsenic change on metabolites.

In the current study of all 610 participants from the FACT trial, we investigated the association of four markers of arsenic exposure (blood AsIII, AsV, MMA, DMA) and three markers of arsenic metabolism (urinary %InAs, %MMA, %DMA) with 11 key OCM metabolites and performed a metabolome-wide association study (MWAS) for both arsenic exposure and arsenic metabolism markers. Furthermore, based on our prior findings that FA supplementation can lower blood arsenic level and facilitate arsenic methylation,^12–15^ we investigated the metabolomic changes that can be attributed to the arsenic change induced by folic acid supplementation.

## Methods

### Study Population and Design

The Folic Acid and Creatine Trial (FACT) is a double-blinded, randomized, placebo-controlled trial designed to investigate the effectiveness of folic acid and creatine supplementation on increasing As methylation and lowering bAs.^12,13^ In 2010, a total of 610 Bangladeshi adults were randomly selected from the Health Effects of Arsenic Longitudinal Study (HEALS) cohort in Araihazar, Bangladesh.^31^ The following selection criteria were applied: (1) 20–75 years of age; (2) had been drinking from their current well with water arsenic >50 μg/L for at least 3 years. We excluded those who were pregnant, had been taking nutritional supplements, or had proteinuria or known renal diseases, diabetes, gastrointestinal or other related health problems. After enrollment, participants were given arsenic-removal water filters and were randomized, separately for men and women, into one of five treatment groups: placebo (PBO, *n* = 102), 400 μg folic acid (400FA, *n* = 153), 800 μg folic acid (800FA, *n* = 150), creatine (CR, *n* = 102), or creatine +400 μg FA (CR+FA, *n* = 103). Follow-up visits were carried out at week 12 and week 24. In the current study, we included information from the baseline on all participants for the cross-sectional analysis, and only included participants in the PBO, 400FA and 800FA arms at week 12 for the change analysis (Figure 1A). From week 12 to 24, half of the participants were switched to placebo and the sample size at week 24 is reduced. Therefore, to obtain the most robust results, this study did not include participants from week 24. Compliance was similar across treatment groups, with an overall compliance of 99.5% as indicated by pill counts.^13^

**Figure 1:**
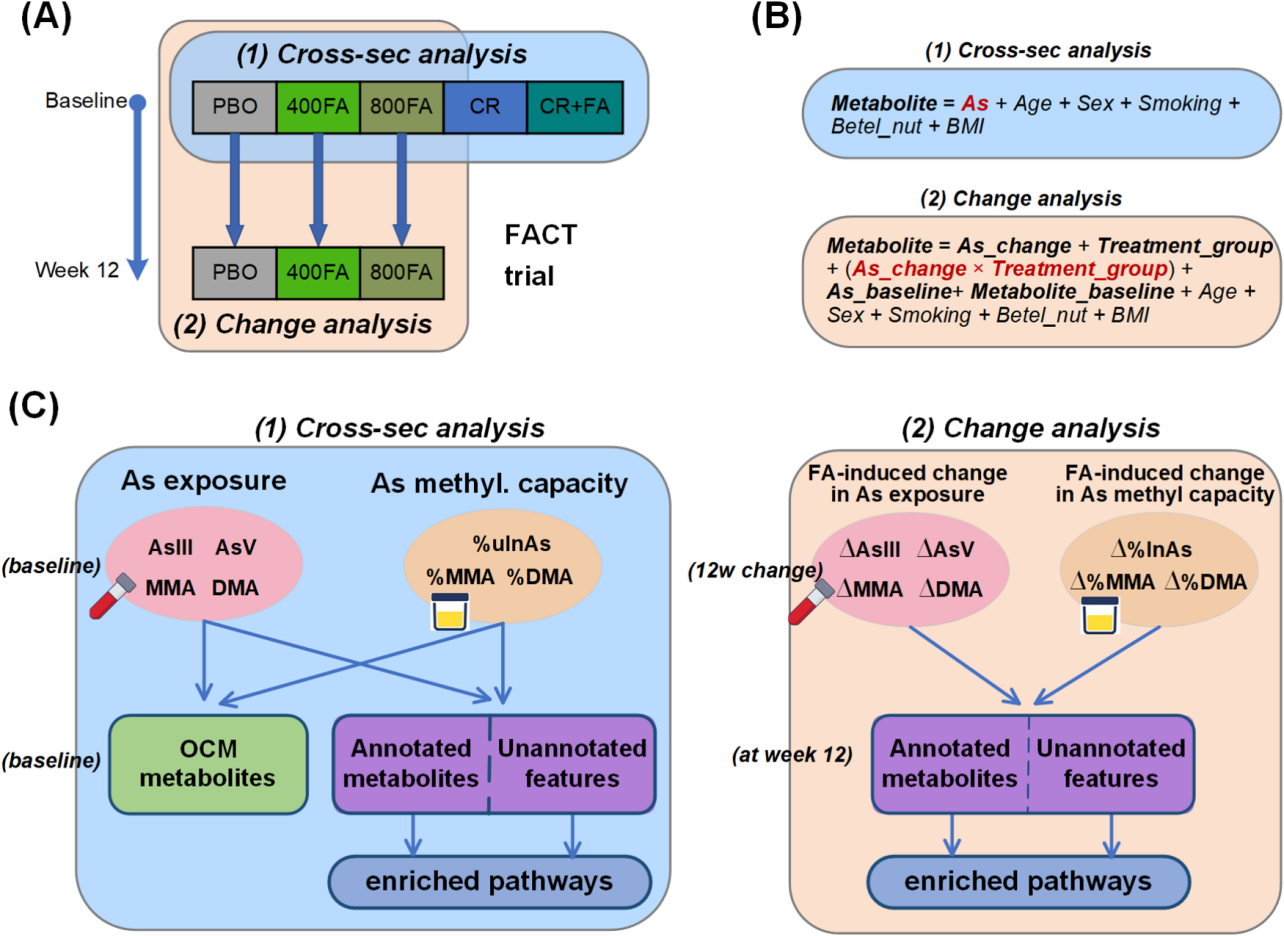
Study design (A), model specification (B), and analytic strategy (C).

Informed consent was obtained by our Bangladeshi field staff physicians. Ethical approval was obtained from the Institutional Review Board of Columbia University Medical Center and the Bangladesh Medical Research Council.

### Assessment of Arsenic, OCM Metabolites, and Metabolomic Profiles

Briefly, blood and urine samples were measured using high performance liquid chromatography (HPLC) coupled to a dynamic reaction cell inductively coupled plasma mass spectrometer. We used two panels of arsenic measurements—four blood arsenic species concentrations (As^III^, As^V^, MMA, and DMA) and three urinary arsenic species percentages (%uInAs, %uMMA, and %uDMA)—to represent arsenic exposure and arsenic metabolism capacity, respectively (Figure 1). Eleven OCM metabolites (including vitamin B12, total homocysteine, cysteine, dimethylglycine, 5-methyl-tetrahydrofolate (5-mTHF), methionine, SAM, S-adenosylhomocysteine (SAH), betaine, choline, and cystathionine) were measured using HPLC-MS/MS. Plasma metabolomic profiles were measured using LC-HRMS. Details on the methodologies can be found in Supporting Information (SI, Supplementary Methods).

### Statistical Analysis

Figure 1 illustrates the overall analytic strategy. We used two sets of arsenic variables (arsenic exposure and arsenic metabolism capacity) and two groups of small metabolite measures (OCM metabolites and plasma metabolome). Statistical analysis was conducted in two parts (Figure 1). First, to identify arsenic-associated OCM metabolites and metabolomic profiles, we performed a cross-sectional analysis using the baseline data from all FACT participants, before randomization and FA supplementation. Next, to identify metabolites that were associated with arsenic change specific to the FA supplementation, we performed a change analysis using both baseline and week 12 data, but only among the PBO (*n* = 99), 400FA (*n* = 150), and 800FA (*n* = 146) groups. Both analyses were conducted using robust linear regression, but with different model specifications as detailed in Supporting Information (SI, Supplementary Methods). False discovery rate (FDR)-adjusted *p* less than 0.05 was considered statistically significant.

Pathway enrichment analyses were performed using the MetaboAnalyst platform (v6.0) for annotated metabolites and the Mummichog for untargeted metabolomic features. Details can be found in Supporting Information (SI, Supplementary Methods).

## Results

### Baseline Characteristics of Study Participants

Twelve participants were excluded due to missing data on blood arsenic (*n* = 3), plasma metabolome (*n* = 3), or BMI (*n* = 6). Table S1 shows the baseline characteristics of the study participants (*n* = 598). We did not observe differences across treatment groups. Overall, the mean age was 38.3 years, 51.0% were male, 27.6% were smokers, 24.6% were betel nut users, and the mean BMI was 19.8 kg/m^2^. For blood arsenic species, the concentrations were the highest for MMA (mean 4.5 μg/L) and the lowest for As^V^ (mean 0.3 μg/L). The bAs^III^-to-bAs^V^ ratio was relatively stable across intervention groups (∼7:1), suggesting that the oxidation of blood As^III^ during sample handling may have minimal or had a uniform effect on the measurement of blood inorganic arsenic. For urinary arsenic species, DMA accounted for the largest proportion (72.6%), and InAs and MMA accounted for 14.3 and 13.1%, respectively. As expected, all blood arsenic species are positively associated with each other and with %uInAs and %uMMA, while %DMA showed negative associations with other arsenic species (Table S2). The concentrations of 11 OCM metabolites were similar among five intervention groups at baseline (Table S3).

### OCM Metabolites Associated with Arsenic Exposure and Metabolism

Using baseline data, we first assessed the association between 11 OCM metabolites and markers of arsenic exposure and metabolism (Figure 2, Table S4). In general, the association patterns were consistent across concentrations of the four bAs species but were different for urinary %InAs compared to %MMA and %DMA. For example, cystathionine was positively associated with all four bAs species. Similarly, cysteine was negatively associated with all 4 bAs species. In contrast, methionine, SAM, and SAH were all negatively associated with %uInAs, and SAM was positively associated with %uDMA. Choline, betaine, and dimethylglycine were negatively associated with %uMMA and positively with %uDMA. We also calculated the sum of bAs^III^, bAs^V^, bMMA, and bDMA, and identified similar metabolites in association with total blood arsenic levels (Figure S1).

**Figure 2:**
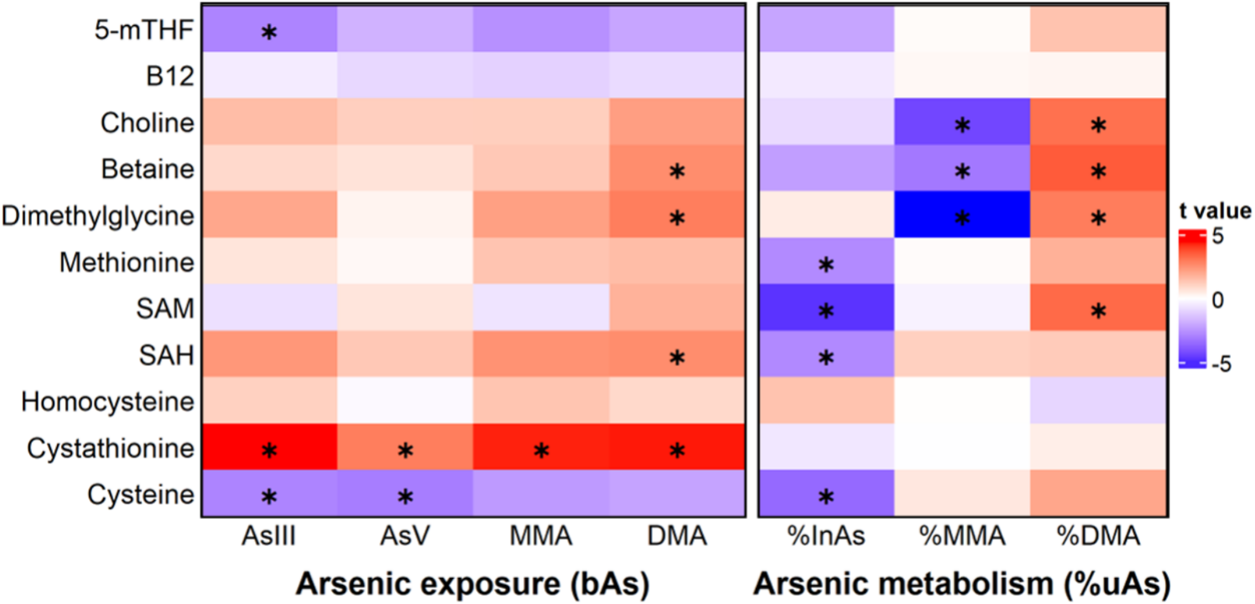
Associations between one-carbon metabolism metabolites and markers of arsenic exposure and metabolism. We used robust linear regression to identify one-carbon metabolism metabolites associated with arsenic exposure (four blood As species) and with arsenic metabolism (three urinary As species percentages) in the trial baseline population, adjusting for age, sex, smoking, betel nut use, and body mass index. Significant associations with FDR < 0.05 were marked with asterisks. *T* values were obtained from regression models and were shown by color. Abbreviations: 5-mTHF, 5-methyl-tetrahydrofolate; AsIII, arsenite; AsV, arsenate; DMA, dimethyl-arsenical species; FA, folic acid; FDR, false discovery rate-adjusted *p* value; InAs, inorganic arsenic; MMA, monomethyl-arsenical species; SAH, S-adenosylhomocysteine; SAM, S-adenosylmethionine.

### Metabolic Profile Associated with Arsenic Exposure

Using data from the trial baseline, we assessed the metabolic profile in association with arsenic exposure (concentrations of blood arsenic species, bAs) at FDR < 0.05 (Figure 3, Table S5). Eight unique bAs-associated metabolites were identified, six of which were associated with As^V^ ([12-methyl-C15.0]-12-methyltetradecanoic acid, 3-Methoxytyrosine, 3-methyl-l-histidine, cystine, orotate, sorbate), three with DMA ([12-methyl-C15.0]-12-methyltetradecanoic acid, 3-hydroxybenzyl alcohol, *N*,*N*,*N*-trimethyllysine), and one with As^III^ ([12-methyl-C15.0]-12-methyltetradecanoic acid; Figure 3A). Three metabolites ([12-methyl-C15.0]-12-methyltetradecanoic acid, 3-hydroxybenzyl alcohol, *N*,*N*,*N*-trimethyllysine) were associated with total blood As at FDR < 0.10, all of which were consistent with the results from As species. We also identified 812 nonannotated metabolomic features in association with bAs, 706 of which were associated with As^V^ (Figure 3B). For both metabolites and nonannotated metabolomic features, we observed predominantly negative associations (72.7 and 86.7% respectively) with arsenic exposure markers. All of the blood As metabolites were above the LOD of 0.22 μg/L except for blood AsV, where 51.3% of observed concentrations were below 0.22 μg/L. We therefore conducted sensitivity analysis to assess the impact of measurement error on AsV result, by replacing values below the LOD with LOD/√2. As illustrated in Figure S2, the significant metabolites were consistently identified among the top hits, suggesting that our findings were robust and not significantly affected by different LOD-handling approaches. In a sensitivity analysis where study participants were grouped by arsenic-methylation efficiency-related genotypes (i.e., 3 SNPs in the AS3MT gene), we did not observe significant interaction at FDR < 0.20, suggesting that our findings may not have been substantially influenced by individual As metabolism capacity (Figure S3).

**Figure 3:**
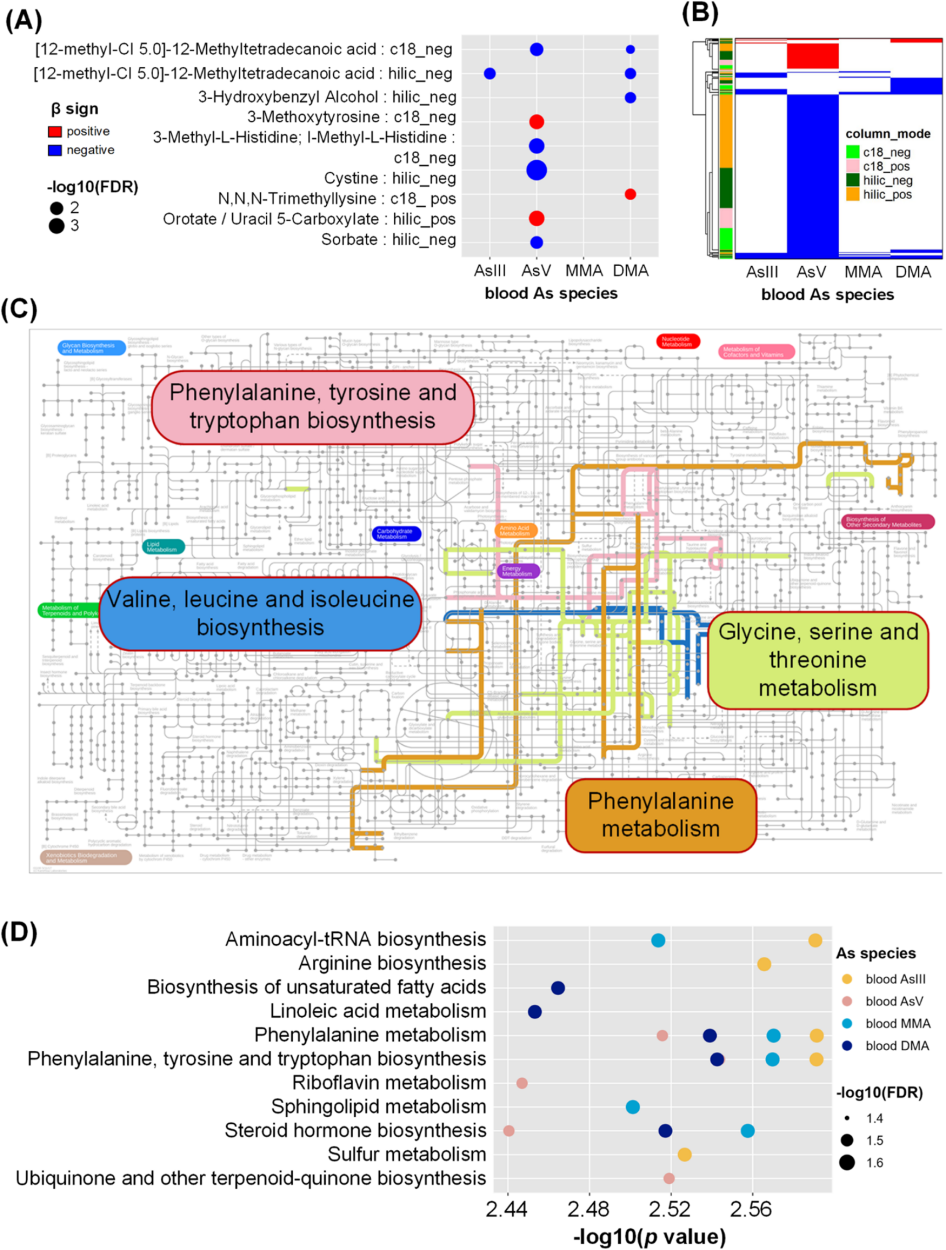
Metabolomic signature of arsenic exposure (blood As) We used robust linear regression to identify annotated metabolites (A) and unannotated metabolic features (B) associated with blood As species (FDR < 0.05) in the trial baseline population, adjusting for age, sex, smoking, betel nut use, and body mass index. Metabolomic profiling uses two column types (c18 and hilic) and two modes (positive and negative), and duplicate metabolites are marked in red in **A**. Pathways significantly enriched at FDR < 0.05 are shown for metabolites (C) and for metabolic features (D). In **D**, due to limited space we showed the top five pathways enriched for each As species for the c18 column using mummichog (FDR < 0.05); the full list can be found in Table S6. Abbreviations: As, arsenic; DMA, dimethyl-arsenical species; FA, folic acid; FDR, false discovery rate-adjusted *p* value; InAs, inorganic arsenic; MMA, monomethyl-arsenical species.

We first performed enrichment analysis for bAs-associated metabolites; four KEGG pathways were enriched at FDR < 0.05 including phenylalanine, tyrosine and tryptophan biosynthesis; phenylalanine metabolism; valine, leucine and isoleucine biosynthesis; and glycine, serine and threonine metabolism (Figure 3C). Figure 3D shows the top 5 pathways enriched for metabolomic features associated with each As species using Mummichog. This enrichment analysis (Figure 3D, Table S6) also highlighted phenylalanine, tyrosine and tryptophan biosynthesis pathway and phenylalanine metabolism pathway, which were significantly enriched for multiple blood arsenic species. In addition, the steroid hormone biosynthesis pathway was also enriched for multiple bAs species. Interestingly, other pathways were unique to only one bAs species.

### Metabolic Profile Associated with Arsenic Metabolism

We further assessed the metabolic profile for arsenic metabolism using baseline data (Figure 4, Table S7). A total of 66%uAs-associated metabolites were identified, 51 of which were associated with %uInAs (Figure 4A). We also identified 285 nonannotated metabolomic features in association with %uAs markers, 199 of which were associated with %uInAs (Figure 4B). We observed some overlap of metabolic profiles between %uInAs and %uDMA, and between %uMMA and %uDMA, and all their associations were in the expected opposite directions. However, there was little overlap between %uInAs- and %uMMA-associated metabolic profiles (Figure 4A,B).

**Figure 4:**
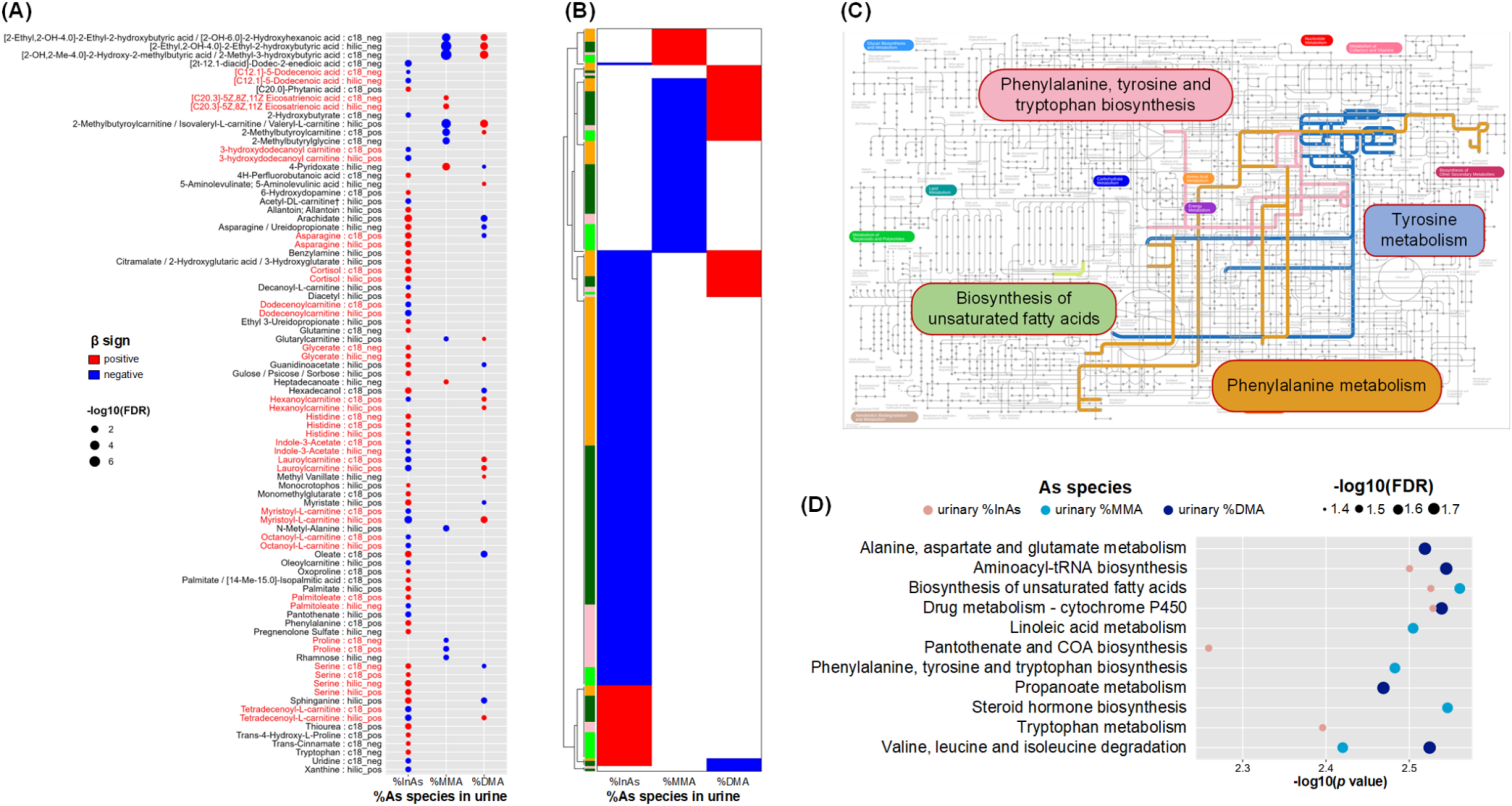
Metabolomic signature of arsenic metabolism (urinary %As) We used robust linear regression to identify annotated metabolites (A) and unannotated metabolic features (B) associated with urinary arsenic species percentage (FDR < 0.05) in the trial baseline population, adjusting for age, sex, smoking, betel nut use, and body mass index. Metabolomic profiling uses two column types (c18 and hilic) and two modes (positive and negative), and duplicate metabolites are marked in red in **A**. Pathways significantly enriched at FDR < 0.05 are shown for metabolites (C) and for metabolic features (D). In **D**, due to limited space we showed the top five pathways enriched for each As species for the c18 column using mummichog (FDR < 0.05); the full list can be found in Table S8. Abbreviations: As, arsenic; DMA, dimethyl-arsenical species; FA, folic acid; FDR, false discovery rate-adjusted *p* value; InAs, inorganic arsenic; MMA, monomethyl-arsenical species.

Four KEGG pathways were enriched for %uAs-associated metabolites, including phenylalanine, tyrosine and tryptophan biosynthesis; phenylalanine metabolism, tyrosine metabolism, and biosynthesis of unsaturated fatty acids pathways (Figure 4C). Enrichment analysis for metabolomic features (Figure 4D, Table S8) also identified phenylalanine, tyrosine and tryptophan biosynthesis pathway and biosynthesis of unsaturated fatty acids pathway. Five of the 11 pathways identified in association with %uAs species were also identified for bAs metabolite concentrations.

### Metabolic Profile Associated with FA-Induced Arsenic Change

Figure 5 and Table S9 show the metabolic profile associated with 12-week changes in arsenic (bAs and %uAs) induced by FA supplementation. We did not identify any metabolites associated with bAs change at FDR < 0.05. However, four metabolites (isopalmitic acid, 3-methylglutaconate, indole-3-aldehyde, oxaloacetic acid) were consistently identified positively associated with urinary %uInAs changes in both 400FA and 800FA groups (FDR < 0.05, Figure 5A). For metabolomic features, a total of 331 features were associated with bAs, of which 312 were associated with As^V^. A total of 927 features were also associated with %uAs, of which 924 were associated with %uInAs (Figure 5B). Notably, there were substantial overlaps between two doses of FA supplementation, especially for %uAs, and the effect directions were consistent across 400FA and 800FA groups.

**Figure 5:**
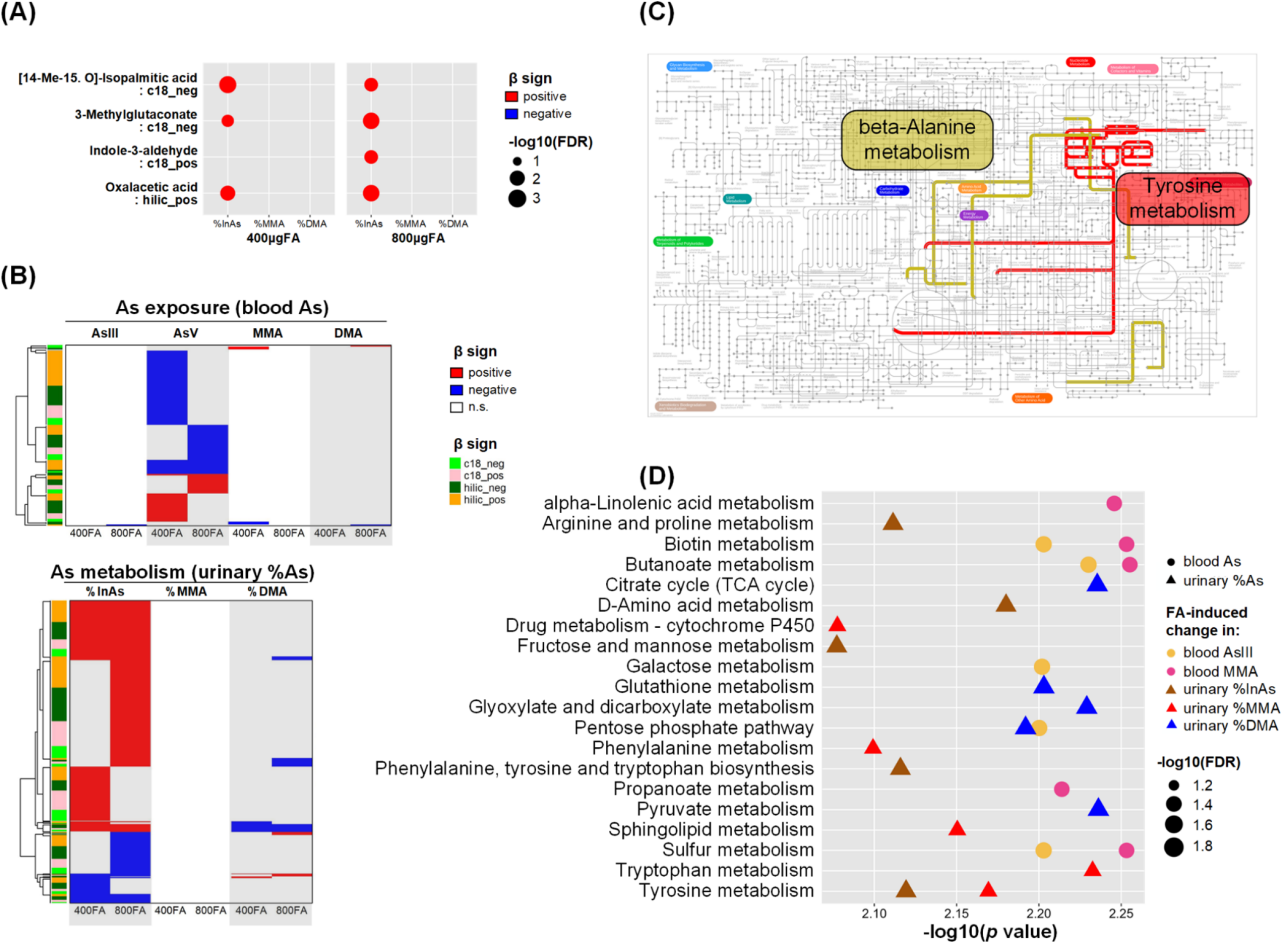
Metabolomic signature of FA-induced arsenic changes during 12-week FA supplementation We used robust linear regression to identify annotated metabolites (A) and unannotated metabolic features (B) associated with changes in blood arsenic species or urinary arsenic species percentage (FDR < 0.05) that are specific to 12-week FA supplementation, adjusting for baseline (at week 0) levels of As and metabolite, intervention groups, age, sex, smoking, betel nut use, and body mass index. For illustration purpose, pathway enrichment analysis was performed combining two doses of FA supplementation groups into one. In **D**, due to limited space we showed the top five pathways enriched for each As species for the c18 column using mummichog (FDR < 0.05); the full list can be found Table S10. Abbreviations: As, arsenic; DMA, dimethyl-arsenical species; FA, folic acid; FDR, false discovery rate-adjusted *p* value; InAs, inorganic arsenic; MMA, monomethyl-arsenical species.

Figure 5C shows the results of pathways enriched for the identified metabolites in association with either bAs or %uAs changes specific to the effect of FA supplementation. For illustration purpose, pathway enrichment analysis was performed combining two doses of FA supplementation groups into one. There are two KEGG pathways (tyrosine metabolism, β-alanine metabolism) significantly enriched at FDR < 0.05 (Figure 5C). For unannotated metabolomic features, pathways were significantly enriched only for features measured on the C18 column. In analyses of the top 5 pathways enriched for each arsenic variable in the change analyses, 7 of the 20 pathways overlapped with one or more of the cross-sectional analyses. For example, sulfur metabolism was associated with inorganic bAs^III^ at baseline and with its change; sphingolipid metabolism was associated with bMMA at baseline and with change in %uMMA. This suggests that these may be arsenic-induced disruptions that could potentially be improved with FA supplementation. The tyrosine metabolism pathway was also enriched, in addition to other metabolic pathways including the phenylalanine, tyrosine and tryptophan biosynthesis and the phenylalanine metabolism pathways (Figure 5D, Table S10).

## Discussion

Arsenic is a multiorgan toxicant and a recognized human carcinogen, yet the broader metabolic implications of arsenic exposure and its metabolism remain to be fully elucidated. This study of adults exposed to moderate to high levels of arsenic characterized the metabolic profiles, including the plasma metabolome and specific OCM metabolites, related to arsenic exposure and metabolism. MWAS identified 8 metabolites associated with blood arsenic concentrations and 66 metabolites associated with arsenic metabolism biomarkers. Leveraging the randomized trial design, we identified four additional metabolites associated with change in %InAs following a 12-week FA supplementation, with findings consistent across two FA doses. In both cross-sectional and change analyses, most of the identified metabolites were associated with As^V^ and %InAs, suggesting that inorganic arsenic significantly impacts the plasma metabolome in this arsenic-exposed population.

OCM supplies methyl groups essential for arsenic methylation. Consistent with findings from randomized controlled trials,^12,13,15^ our baseline cross-sectional analyses identified 5 mTHF to be negatively associated with concentrations of bAs^III^, bMMA and bDMA (*ps* < 0.05). In the cross-sectional analyses at baseline, methionine, SAM, and SAH were negatively associated with %uInAs, which is anticipated given their roles in OCM. Our findings also corroborate previous reports that betaine, an alternative methyl donor, and its precursor choline may enhance arsenic methylation capacity^32^ as they have been linked to decreased %MMA^33^ and increased %DMA in urine.^33,34^ Our analysis confirmed these findings and also observed a positive association between betaine and blood DMA levels. The association between choline/betaine and arsenic methylation capacity may be particularly pronounced in populations having marginal folate status.^33^ Finally, in line with previous findings,^35^ we observed a negative association of cysteine with both blood and urinary InAs in the OCM analysis. These findings align with the understanding that cysteine may enhance arsenic detoxification by promoting reduction of pentavalent arsenicals, a key step to facilitate As methylation, through its role in glutathione biosynthesis.^36^

Interestingly, we found that plasma concentrations of cystathionine, an intermediate in the transsulfuration pathway and thiol metabolism, were positively associated with concentrations of all four blood arsenic metabolites. The transsulfuration pathway is important: (1) for the transfer of sulfur from methionine to cysteine; (2) for lowering homocysteine and SAH; and (3) for generating glutathione; all these roles may be related to blood arsenic concentrations. For example, SAH inhibits the methylation and urinary elimination of arsenic,^37^ and glutathione stimulates both arsenic metabolism and efflux from cells.^38^ Although to our knowledge no study has directly assessed the association between arsenic exposure and cystathionine levels, studies suggested that arsenic can alter cystathionine-ß-synthase (CBS), the enzyme that synthesizes cystathionine, in animal models.^39,40^ Given that CBS is a redox-sensitive enzyme, arsenic-induced oxidative stress may increase CBS activity. In addition, trivalent arsenicals have a high affinity for thiols causing inhibition of several enzymes.^41^ This has been confirmed in our pathway analysis where sulfur metabolism was enriched for bAs^III^. Interestingly, higher plasma concentrations of cystathionine have been associated with increased cardiovascular and noncardiovascular mortality in large cohorts of CHD patients.^42^ Future studies are needed to evaluate if cystathionine may be a potential mediator between arsenic exposure and risk for arsenic-related health outcomes.

There is mounting evidence indicating that arsenic exposure influences the metabolome. A review in 2022 summarized 12 publications on arsenic-related metabolomics studies in humans, of which only three were conducted in general populations and they all assessed the metabolome in urine. Two small studies of the urinary metabolome found that %InAs^29^ and total urinary arsenic^27^ were associated with urinary serine, which is consistent with our findings where %uInAs was consistently associated with serine across four different MS column types and modes. The third study conducted in a Polish population living in the vicinity of a copper smelter area reported urinary metabolome profile,^28^ but the main arsenic species driving the difference in total uAs was arsenobetaine. Arsenobetaine was measured in FACT but we excluded it from our data analyses because it is a nontoxic form of arsenic found in seafood. Recently, a study of 1992 Chinese adults^43^ reported significant associations between plasma arsenic levels and 39 plasma metabolites enriched for biosynthesis of unsaturated fatty acids and linoleic acid metabolism pathways, which were confirmed in our study as the top enriched pathways for both arsenic exposure and metabolism markers.

Our findings are consistent with those of our pilot study.^30^ For example, eicosatrienoic acid (20:3n-3 fatty acid) is positively associated with %MMA in both studies.^30^ Omega-3 polyunsaturated fatty acids are known to protect against skin lesions^44^ and a previous study showed that eicosatrienoic acid is elevated in patients with skin lesions induced by chronic arsenic exposure.^18^ Thus, eicosatrienoic acid might contribute to the metabolic response of arsenic in the skin. Another example is proline which was negatively associated with %MMA. Proline is an amino acid essential for collagen biosynthesis and skin health,^45^ and can act as a metal chelator and prevent oxidative damage.^46^ Thus, proline may play a role in alleviating arsenic-induced oxidative stress and skin lesions. One of the intriguing findings from both analyses is that most of the identified metabolites were associated with blood As^V^. There is a scarcity of comparable results in the literature because previous studies usually analyze As^V^ and As^III^ together as total inorganic arsenic; however, it is known that inorganic As^V^ (AsO_4_^3–^) shares structural similarity with phosphate (PO_4_^3–^) and can substitute for phosphate in biochemical reactions.^47^ Future studies with bAs^V^ measurements are warranted to confirm these findings.

In this study, the phenylalanine, tyrosine and tryptophan biosynthesis and metabolism-related pathways have been identified for both arsenic exposure and metabolism markers, in both metabolite- and metabolomic feature-oriented analyses, consistent with evidence from other populations and rodent models.^19,48–50^ Three component metabolites defining this pathway, phenylpyruvic acid, phenylalanine, tyrosine, were found nominally significant in their associations with blood arsenic species, and phenylalanine was significantly associated with urinary %InAs after FDR correction. Phenylalanine and tyrosine serve as precursors for melanin. Melanosis, a form of skin hyperpigmentation associated with increased melanin, is the hallmark of chronic arsenic exposure.^51,52^ Although the exact mechanism is not clear, increased melanin synthesis induced by arsenic exposure has been suggested as one crucial mechanism.^53^ Dysregulation of phenylalanine and tyrosine metabolism may also cause cancer and neurodegenerative disease.^54^ We observed a positive association between %InAs and tryptophan.^30^ Tryptophan functions as a precursor for serotonin, melatonin, kynurenine, and niacin, and contributes to coordinating immune homeostasis.^55,56^ Tryptophan dysregulation has been linked with many arsenic-related diseases.^57^ Furthermore, while phenylalanine and tryptophan are distinct amino acids with unique roles and metabolic pathways, they are both metabolized through the hepatic kynurenine pathway, and are both precursors for neurotransmitters, highlighting their potential roles in arsenic-related neurological effects. However, more research is needed to fully understand the mechanisms and extent of the identified associations.

In the FACT study, FA treatment decreased %uInAs. In this study, we identified four metabolites ([14-Me-15.0]-isopalmitic acid, oxaloacetic acid (OAA), 3-methylglutaconate and indole-3-aldehyde) in association with FA-induced change in urinary %InAs over 12 weeks of FA supplementation, and the findings were consistent across two FA doses. Three of these share links to acetyl-CoA, a molecule that participates in many biochemical reactions but with the main function of delivering an acetyl group to the citric acid cycle for energy production. The first, a palmitic acid (PA) metabolite, was also positively associated with %uInAs in our baseline cross-sectional analyses. PA is a major component of the human fat depot^58^ and was associated with BMI in our study (data not shown). PA inhibits the conversion of acetyl-CoA to malonyl-CoA. The second, OAA reacts with acetyl-CoA to form citrate in the citric acid cycle.^59^ OAA also influences insulin signaling.^60^ The third, 3-methylglutaconate, is an intermediate in the mevalonate shunt, a pathway that is also linked to acetyl-CoA metabolism. Arsenite is known to inhibit the conversion of pyruvate to acetyl-CoA through binding to the lipoic acid cofactor within the pyruvate dehydrogenase (PDH) complex.^61^ Thus, while no evidence has directly linked these 3 metabolites with arsenic or folate, the data may be capturing effects that are proximal to arsenite-induced PDH inhibition and may be alleviated by FA-induced reductions in %InAs.

In addition to metabolites, we also identified significant pathways for FA-induced arsenic change, one of which is tyrosine metabolism. Interestingly, one key metabolite of tyrosine metabolism is thyroxine, which has been linked with arsenic exposure in both Bangladeshi and Chinese populations,^62,63^ and was also found nominally associated with %uInAs in our study. Tyrosine metabolism is also responsible for generating neurotransmitters such as dopamine and norepinephrine, which are known to be influenced by OCM and may have implications for neurodegenerative diseases. Together, this evidence suggests that FA-induced changes in %uInAs affect tyrosine metabolism and may alter the risks of multiple arsenic-related health conditions.

Our study has several strengths. It is one of the largest studies to date that employs high-resolution MS to comprehensively assess the plasma metabolome related to arsenic, and we considered markers for both arsenic exposure and arsenic metabolism capacity. We additionally examined arsenic-related OCM metabolites, which complemented and corroborated our MWAS findings. Utilizing the trial design, we provided the first evidence on the metabolomic profile of the arsenic change induced by FA supplementation, and the findings were consistent across two FA doses. However, our study was established among Bangladeshi adults with chronic exposure to moderate to high levels of arsenic, thus the generalizability of our findings may be limited in populations of other genetic backgrounds, ages, and exposure routes and levels. Given the trial design (i.e., only half of the FA treatment group participants continued on FA through week 24) and data availability, we only examined the 12-week effect of folic acid on arsenic metabolism; this duration is unlikely to capture long-term health outcomes. Finally, our results should be interpreted cautiously as despite the original trial design, the associations of arsenic exposure and FA-induced arsenic reduction with metabolic features and pathways are cross-sectional.

In this MWAS analysis, we identified different metabolites and pathways associated with arsenic exposure, arsenic metabolism, and the 12-week metabolomic changes induced by FA supplementation. Most of the identified metabolites were associated with blood As^V^ and with urinary %InAs, highlighting the impact of inorganic arsenic on metabolic processes. Most of the arsenic-associated metabolites were enriched for pathways related to phenylalanine, tyrosine and tryptophan biosynthesis and metabolism; links between alterations in these metabolites and development of melanosis warrant further study. In analyses of the top pathways enriched for each arsenic variable in the FA-induced change analyses, several pathways overlapped with one or more of the cross-sectional analyses. This suggests that these may be arsenic-induced disruptions that could potentially be improved with FA supplementation. Collectively, these findings may enhance our understanding of the mechanisms underlying arsenic-induced health outcomes and could ultimately inform prevention and treatment strategies to mitigate the adverse effects of arsenic exposure.

## Supplementary Material





## Notes

Associations between one-carbon metabolism metabolites and markers of arsenic exposure and metabolism, including results for total blood arsenic; sensitivity analysis for blood As^V^-associated metabolites—replacing values < LOD with LOD divided by square root of 2; sensitivity analysis – significant associations between blood arsenic species and metabolites identified in Figure 3 stratified by As methylation efficiency (AME) status (PDF)
Baseline characteristics of study participants; Spearman correlation between bAs and %uAs species; one-carbon metabolism metabolites across treatment groups in the baseline population; association between arsenic species and OCM metabolites in the baseline population; metabolome-wide association study of blood arsenic species in the baseline population (*P* < 0.05); pathway enrichment analysis for untargeted features in association with blood arsenic species; metabolome-wide association study of urinary arsenic species percentage in the baseline population (*P* < 0.05); pathway enrichment analysis for untargeted features in association with urinary arsenic species percentage; metabolome-wide association study of arsenic change induced by folic acid supplementation over 12-week intervention (*P* < 0.05 in either dose); pathway enrichment analysis for untargeted features in association with arsenic change induced by folic acid supplementation over 12-week intervention (XLSX)

## ††

Department of Biochemistry and Molecular Biology, Mawlana Bhashani Science and Technology University, Tangail, Bangladesh
