# Supplementary material for: Metabolic Signature of Arsenic Exposure and Metabolism: The Folic Acid and Creatine Trial
Source: Environ Sci Technol. 2025 Jul 16;59(29):14905–16. doi: 10.1021/acs.est.5c01597 (PMC12312088; doi:10.1021/acs.est.5c01597)
Supplement: Supplementary file 1 [file es5c01597_si_001.pdf]

*Supporting Information*

# Metabolic signature of arsenic exposure and metabolism: the Folic Acid and Creatine Trial

Wending Li[1], Haotian Wu[1], Jeff Goldsmith[2], Ronald A Glabonjat[1], Vesna Ilievski[1], Olgica Balac[1], Vesna Slavkovich[1], Brismar Pinto-Pacheco[3], Xiangping Lin[3,4], Faruque Parvez[1], Gabriela L Jackson[1], Abu B Siddique[5], Mohammad Nasir Uddin[5,#], Tariqul Islam[5], Irene Martinez-Morata[1], Ana Navas-Acien[1], Megan M Niedzwiecki[3], Marianthi-Anna Kioumourtzoglou[1], Brandon L Pierce[6,7,8], Joseph H Graziano[1], Teodoro Bottiglieri[9], Douglas I Walker[10], Mary V Gamble[1,*]

[1]Department of Environmental Health Sciences, Mailman School of Public Health, Columbia University, New York, NY, 10032, USA
[2]Department of Biostatistics, Mailman School of Public Health, Columbia University, New York, NY, 10032, USA
[3]Department of Environmental Medicine and Climate Science, Icahn School of Medicine at Mount Sinai, New York, NY, 10029, USA
[4]Department of Genetics, Stanford University School of Medicine, Stanford, California, 94305, USA
[5]Columbia University Arsenic Project in Bangladesh, Dhaka, Bangladesh
[6]Department of Public Health Sciences, University of Chicago, Chicago, IL, 60637, USA
[7]Department of Human Genetics, University of Chicago, Chicago, IL, 60637, USA
[8]Comprehensive Cancer Center, University of Chicago, Chicago, IL, 60637, USA
[9]Center of Metabolomics, Institute of Metabolic Disease, Baylor Scott and White Research Institute, Dallas, TX, 75226, USA
[10]Gangarosa Department of Environmental Health, Rollins School of Public Health, Emory University, Atlanta, GA, 30322, USA
[#] Present address: Department of Biochemistry and Molecular Biology, Mawlana Bhashani Science and Technology University, Tangail, Bangladesh

Corresponding Author:

M.V. Gamble, Department of Environmental Health Sciences, Mailman School of Public Health, Columbia University, 722 West 168th St., Room 1107E, New York, NY 10032 USA. Telephone: (212) 305-7949. E-mail: mvg7@cumc.columbia.edu

## Table of Contents

**Supplementary Methods**

**Figure S1.** Associations between one-carbon metabolism metabolites and markers of arsenic exposure and metabolism, including results for total blood arsenic.

**Figure S2.** Sensitivity analysis for blood $As^V$-associated metabolites – replacing values <LOD with LOD divided by square root of 2.

**Figure S3**. Sensitivity analysis – significant associations between blood arsenic species and metabolites identified in **Figure 3** stratified by As methylation efficiency (AME) status.

**Table S1-S9 provided in a separate Excel file.**

**Summary: 9 pages, 3 figures, 0 table**

## Supplementary Methods

*Assessment of blood and urine arsenic*

Blood and urine samples that were used for these analyses were collected at baseline and week 12. Aliquots of ethylenediaminetetraacetic acid-anticoagulated whole blood and plasma samples were stored at –80°C and shipped on dry ice. Urine samples were collected in 50-mL acid-washed polypropylene tubes, stored at –20°C, and shipped on dry ice.[1] For both blood and urine samples, we measured arsenic species ($As^{III}$, $As^{V}$, MMA, and DMA) using high performance liquid chromatography (HPLC) coupled to a dynamic reaction cell inductively coupled plasma mass spectrometer. The detection limits (LOD) and precision of arsenic measurements can be found in our prior publications.[2, 3] The concetrations for all arsenic species were above LOD except $As^{V}$, for which 51.3% were below the LOD of 0.22 μg/L. In accordance with previous research, we employed all data points, including those below the LOD, for our main analysis, as this approach has demonstrated superior performance compared to other LOD-handling methods.[4] To assess the robustness of our findings, we conducted sensitivity analyses by comparing the beta coefficients and p values to those with concentrations below the LOD replaced with $LOD/\sqrt{2}$.

As described previously,[5] arsenic species in urine are susceptible to oxidation (from $As^{III}$ to $As^{V}$) during storage and sample collection and preparation. We therefore calculated the sum of $As^{III}$ and $As^{V}$ levels to represent total inorganic arsenic (ΣInAs) in urine. In addition to the absolute exposure levels, arsenic metabolism capacity (assessed by the relative proportions of arsenic species in urine, %uAs) also varies across individuals and modifies future disease risk from arsenic exposure.[6, 7] Therefore, we used two panels of arsenic measurements – four blood arsenic species concentrations ($As^{III}$, $As^{V}$, MMA, and DMA) and three urinary arsenic species percentages (%uInAs, %uMMA, and %uDMA) – to represent arsenic exposure and arsenic metabolism capacity, respectively (**Figure 1**).

*Assessment of OCM metabolites*

As previously described,[8] plasma vitamin B12 was measured using a radio protein-binding assay (SimulTRAC-SNB; MP Biomedicals), with intra- and inter-assay coefficients of variation (CVs) of 6% and 17%, whereas plasma total homocysteine and cysteine concentrations were measured by HPLC with fluorescence detection using the method of Pfeiffer et al,[9] with intra- and inter-assay CVs of 4% and 7% and of 5% and 8%, respectively. Plasma dimethylglycine concentrations were measured using HPLC coupled with tandem mass spectrometry (MS/MS) at Division of Nutritional Sciences Cornell University, with overall intra- and inter-assay CVs less than 10%.[10, 11] The analyses of 5-methyl-tetrahydrofolate (5-mTHF) and other OCM intermediates, including methionine, SAM, S-adenosylhomocysteine (SAH), betaine, choline, and cystathionine, were carried out at the Center of Metabolomics, Baylor Scott & White

Research Institute using HPLC-MS/MS-based methods,[12, 13] with overall intra- and inter-assay CVs of less than 15%. Three samples with missing data on OCM metabolites were excluded from downstream analyses.

*High-resolution metabolomic profiling and data processing*

Metabolomic profiling was performed for plasma samples collected at baseline, week 12, and week 24. The method has been described in detail in a prior publication.[14] Briefly, plasma samples were prepared in batches of up to 84 study samples containing up to 12 QA/QC samples using an automated liquid handler (Opentron OT2) and 96-well plates. Prior to analysis, samples were thawed at 4ºC, vortexed and 30 μL plasma was extracted by adding 90 μL acetonitrile containing $^{13}C$ labeled internal standards (ISs). The resulting extracts were analyzed using two separate analytical runs configured for $C_{18}$ or HILIC analysis that included a Vanquish Duo Ultra Performance Liquid Chromatography coupled to Q-Exactive HFX HRMS system (Thermo Fisher Scientific, Rockford, IL, USA). Samples were randomized and analyzed using dual column chromatography with mobile phases optimized for positive or negative ionization. All samples were analyzed with reverse phase Higgins TARGA $C_{18}$ 5μm 50x2.1mm column (Higgins Analytical, Inc, Mountain View, CA, USA) in both positive mode and negative mode. HILIC chromatography separation was accomplished using a SeQuant ZIC-HILIC 3.5μm 50x4.6mm column (Merck KGaA, Darmstadt, Germany) for positive mode and a XBridge Amide 3.5μm 3.0x50mm column for negative mode. The mass spectrometer was operated at 120,000 resolution and mass scan range of 85-1250 mass-to-charge ratio (m/z). Raw data was processed through apLCMS [15-17] and xMSanalyzer,[18] which resulted in a total of 20,758 C18 and 31,386 HILIC metabolomic features. ComBat [19] was used to correct for batch effect. To identify known metabolites (Level 1 confidence)[20] we compared detected feature m/z and retention time to an in-house library of metabolite standards run on the same platform and analytical method, which identified 180 C18+, 221 C18-, 223 HILIC+, and 173 HILIC- confirmed metabolites. We also used xMSannotator with the HMDB database to assign predicted metabolite annotations for unconfirmed features.[21, 22]

Prior to analysis, we removed metabolites or metabolomic features with detection rates < 50%, which resulted in a total of 171 C18+, 192 C18-, 203 HILIC+, and 157 HILIC- metabolites, and 9,446 C18+, 7,410 C18-, 12,461 HILIC+, and 12,058 HILIC- features. Since some metabolites can be readily detected by multiple columns and MS modes, a total of 511 unique metabolites were used for analysis. However, we retained the duplicate metabolites in statistical analysis to test whether same metabolites can be consistently identified across different column types and modes. We replaced values below limits of detection with the lowest observed values divided by square root of 2, quantile normalized and log2 transformed the data prior to modeling.

*Additional detail on statistical analysis*

To identify metabolites associated with arsenic, we used robust linear regression to identify OCM metabolites, annotated metabolites, and unannotated features associated with blood arsenic

or urinary %As, adjusting for age, sex, history of smoking (yes/no), betel nut use (yes/no), and BMI (**Figure 1B**).

To identify metabolites associated with FA-induced arsenic change, we calculated the differences in arsenic species between baseline ($t_0$) and first follow-up at week 12 ($t_1$) and constructed the following robust linear regression model (**Figure 1B**):

$$Metabolite_{t1} = \beta_1 \cdot As\ species_{t1-t0} + \beta_2 \cdot (As\ species_{t1-t0} \times Treatment\ group) + \beta_3 \cdot Treatment\ group + \beta_4 \cdot As\ species_{t0} + \beta_5 \cdot Metabolite_{t0} + \beta_6 \cdot Age_{t0} + \beta_7 \cdot Sex_{t0} + \beta_8 \cdot Smoking_{t0} + \beta_9 \cdot Betel\ nut_{t0} + \beta_{10} \cdot BMI_{t0}$$

In the above model, $As\ species_{t1-t0}$ is the difference in arsenic species levels between $t_0$ and $t_1$, $As\ species_{t0}$ is the arsenic species level at $t_0$, $Metabolite_{t0}$ is the metabolite level at $t_0$, and $Metabolite_{t1}$ is the metabolite level at $t_1$. For the '*Treatment group*' variable, we set the placebo group as the referent and generated two dummy variables (for the 400FA and 800FA groups respectively). Thus, the model controls for any temporal, non-treatment effect of arsenic change (e.g., use of water arsenic filters, seasonal change, etc.) as captured in the placebo group ($\beta_1$), while estimating treatment-specific effects of arsenic change through $\beta_2 s$. In addition, the model also controlled for treatment ($\beta_3$), baseline arsenic ($\beta_4$), baseline metabolite ($\beta_5$), and other covariates including age, sex, smoking, betel nut use and BMI ($\beta_{6-10}$).

In a post hoc sensitivity analysis, we explored whether the observed significant associations may differ by As methylation efficiency (AME) status as defined by previously reported AME-related single nucleotide polymorphisms (SNPs).[23] Genome-wide profiling was performed using the Infinium Global Diversity Array. Of the four AME related SNPs, one *AS3MT* SNP, rs11191492, did not pass the data QC, and the other three AME-related SNPs were included in this analysis: rs4919690, rs191177668, and rs61735836. A total of 583 participants with both genotyping data and meta data available were grouped according to whether or not they had at least one mutant allele for any of the 3 SNPs.

*Pathway analysis*

We used the global test algorithm implemented by the Enrichment Analysis module on the MetaboAnalyst platform (v6.0) for the enrichment of annotated metabolites with nominal $p < 0.05$ due to limited numbers of significant hits.[24] To enrich pathways for metabolomic features, we used Mummichog with mixed mode, 5 parts per million mass tolerance, top 5% peaks as p-value cutoff, minimum library size at 3; peak information including m/z, retention time, and p value and z score from regression, as implemented by the MetaboAnalyst R package (v4.0.0).[25] We used FDR-adjusted $p$ values derived from Gamma distribution to determine significant pathways. Both enrichment analyses used Kyoto Encyclopedia of Genes and Genomes (KEGG) metabolic pathway set as the reference library. We used iPATH [26] to visualize the identified metabolites and their enriched KEGG pathways in the form of a metabolic map.

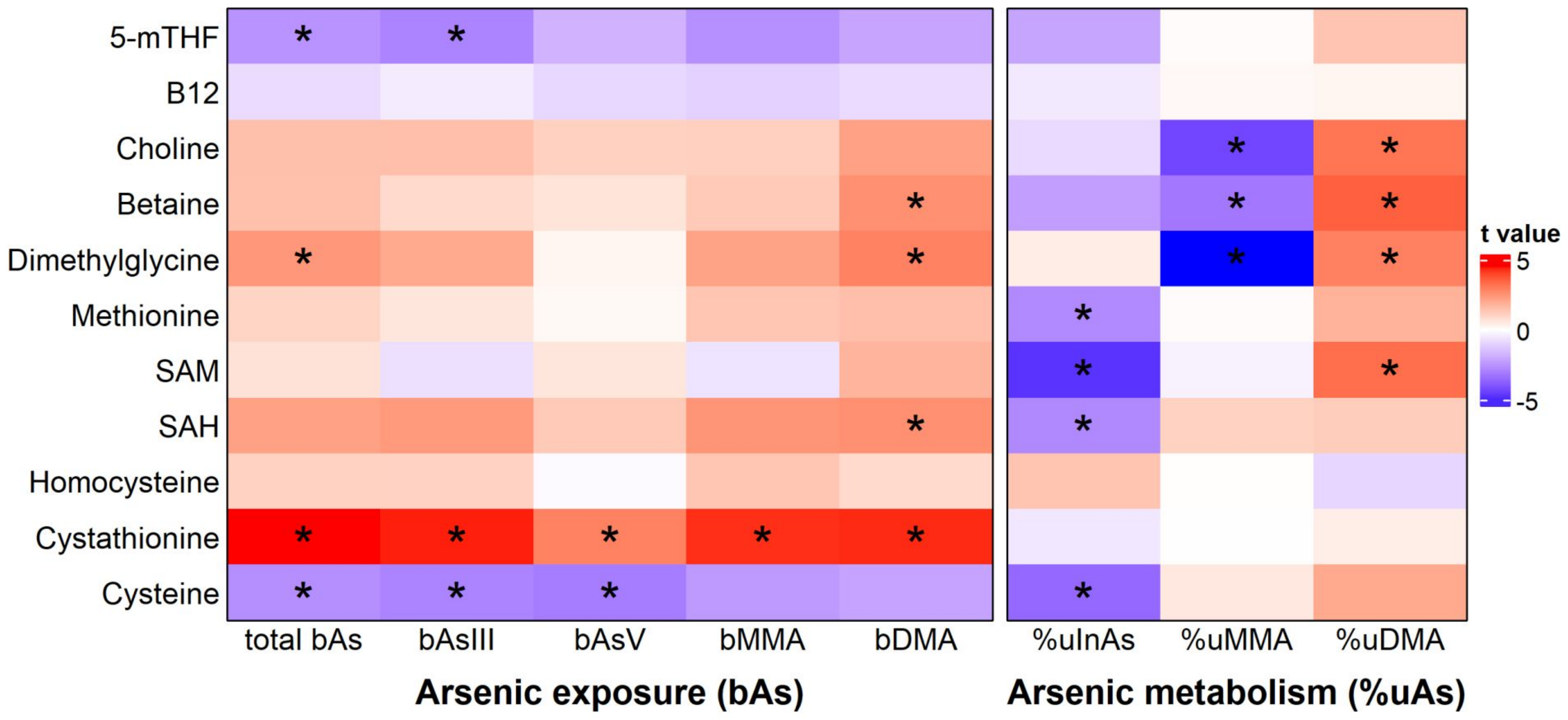


**Figure S1.** Associations between one-carbon metabolism metabolites and markers of arsenic exposure and metabolism, including results for total blood arsenic**.**

We used robust linear regression to identify one-carbon metabolism metabolites associated with arsenic exposure (four blood As species) and with arsenic metabolism (three urinary As species percentages) in the trial baseline population, adjusting for age, sex, smoking, betel nut use, and body mass index. Significant associations with FDR < 0.05 were marked with asterisks. t values were obtained from regression models and were shown by color.

Abbreviations: 5-mTHF, 5-methyl-tetrahydrofolate; AsIII, arsenite; AsV, arsenate; DMA, dimethyl-arsenical species; FA, folic acid; FDR, false discovery rate-adjusted *p* value; InAs, inorganic arsenic; MMA, monomethyl-arsenical species; SAH, S-adenosylhomocysteine; SAM, S-adenosylmethionine.

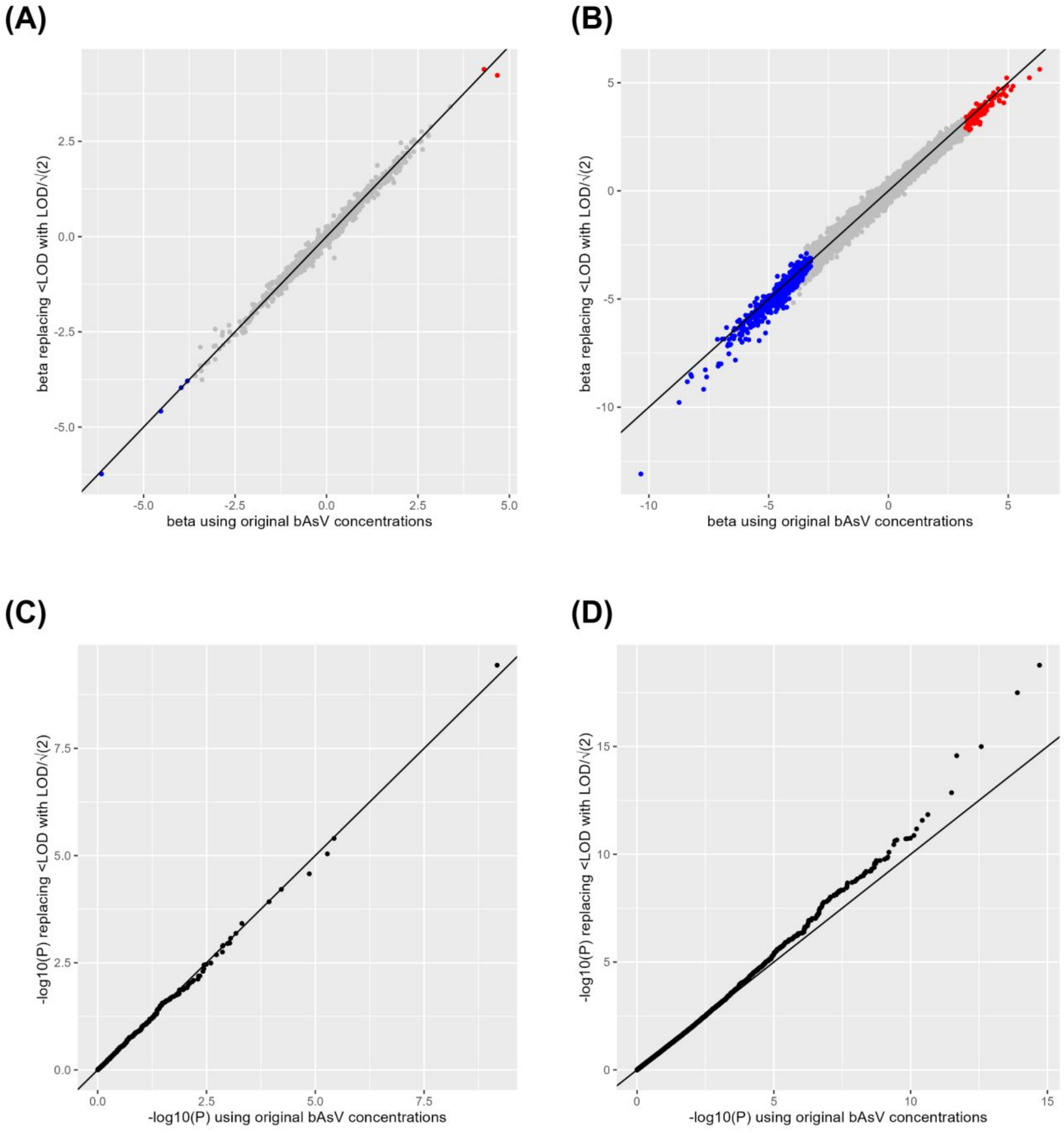


**Figure S2.** Sensitivity analysis for blood $As^V$-associated metabolites – replacing values <LOD with LOD divided by square root of 2.

Robust linear regression was used to identify $As^V$-associated metabolites adjusting for age, sex, smoking, betel nut use, and body mass index. Metabolome-wide results using original $As^V$ concentrations were compared to the results replacing values <LOD with LOD divided by square root of 2. Beta coefficients were compared directly for the same metabolites (**A**) or metabolomic features (**B**), with significant results (**Figure 3**) highlighted in blue (negative) or red (positive). Distributions of *P* values were also compared for metabolites (**C**) or metabolomic features (**D**) using Q-Q plot.

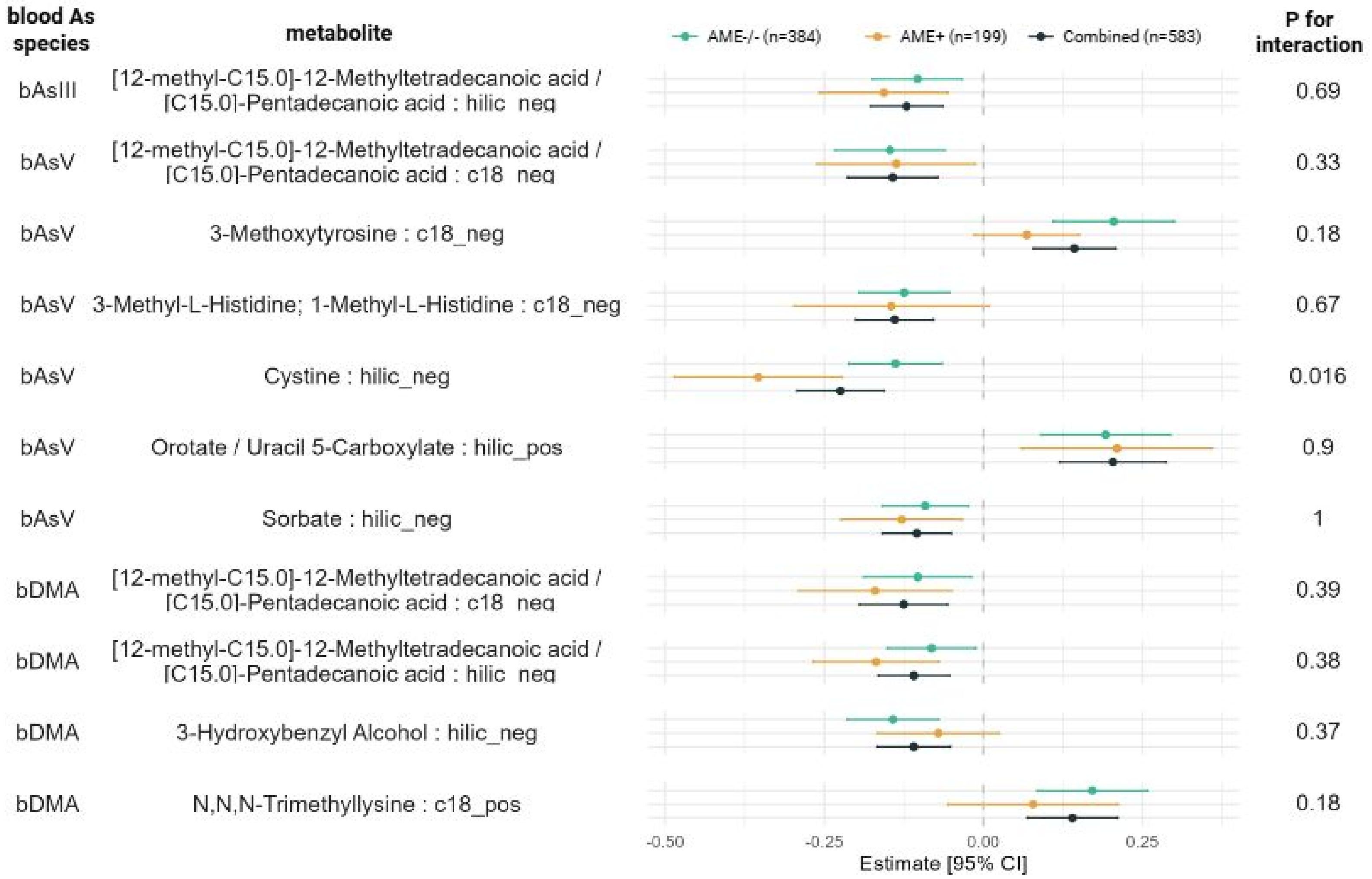


**Figure S3**. Sensitivity analysis – significant associations between blood arsenic species and metabolites identified in **Figure 3** stratified by As methylation efficiency (AME) status. In 583 participants with single nucleotide polymorphisms (SNPs) data available, we used three established AME-related SNPs (rs4919690, rs191177668, rs61735836) to define participants with differential AME capacity: no mutation (AME-/-) or at least one mutant allele for any of the 3 SNPs (AME+). The association between bAsV and Cystine reached nominal significance (P=0.02) but did not pass false discovery rate adjustment (FDR=0.17).

**References**


1. Peters, B. A.; Hall, M. N.; Liu, X.; Parvez, F.; Sanchez, T. R.; van Geen, A.; Mey, J. L.; Siddique, A. B.; Shahriar, H.; Uddin, M. N.; Islam, T.; Balac, O.; Ilievski, V.; Factor-Litvak, P.; Graziano, J. H. Gamble, M. V. Folic Acid and Creatine as Therapeutic Approaches to Lower Blood Arsenic: A Randomized Controlled Trial. *Environ. Health Perspect.* **2015,** *123* (12), 1294-301.
2. Abuawad, A. K.; Bozack, A. K.; Navas-Acien, A.; Goldsmith, J.; Liu, X.; Hall, M. N.; Ilievski, V.; Lomax-Luu, A. M.; Parvez, F.; Shahriar, H.; Uddin, M. N.; Islam, T.; Graziano, J. H. Gamble, M. V. The Folic Acid and Creatine Trial: Treatment Effects of Supplementation on Arsenic Methylation Indices and Metabolite Concentrations in Blood in a Bangladeshi Population. *Environ. Health Perspect.* **2023,** *131* (3), 37015.
3. Bozack, A. K.; Hall, M. N.; Liu, X.; Ilievski, V.; Lomax-Luu, A. M.; Parvez, F.; Siddique, A. B.; Shahriar, H.; Uddin, M. N.; Islam, T.; Graziano, J. H. Gamble, M. V. Folic Acid Supplementation Enhances Arsenic Methylation: Results from a Folic Acid and Creatine Supplementation Randomized Controlled Trial in Bangladesh. *Am. J. Clin. Nutr.* **2019,** *109* (2), 380-391.
4. Keizer, R. J.; Jansen, R. S.; Rosing, H.; Thijssen, B.; Beijnen, J. H.; Schellens, J. H. Huitema, A. D. Incorporation of Concentration Data Below the Limit of Quantification in Population Pharmacokinetic Analyses. *Pharmacol Res Perspect* **2015,** *3* (2), e00131.
5. Feldmann, J.; Lai, V. W.; Cullen, W. R.; Ma, M.; Lu, X. Le, X. C. Sample Preparation and Storage Can Change Arsenic Speciation in Human Urine. *Clin. Chem.* **1999,** *45* (11), 1988-97.
6. Abuawad, A.; Bozack, A. K.; Saxena, R. Gamble, M. V. Nutrition, One-Carbon Metabolism and Arsenic Methylation. *Toxicology* **2021,** *457*, 152803.
7. Bozack, A. K.; Saxena, R. Gamble, M. V. Nutritional Influences on One-Carbon Metabolism: Effects on Arsenic Methylation and Toxicity. *Annu. Rev. Nutr.* **2018,** *38*, 401-429.
8. Bozack, A. K.; Howe, C. G.; Hall, M. N.; Liu, X.; Slavkovich, V.; Ilievski, V.; Lomax-Luu, A. M.; Parvez, F.; Siddique, A. B.; Shahriar, H.; Uddin, M. N.; Islam, T.; Graziano, J. H. Gamble, M. V. Betaine and Choline Status Modify the Effects of Folic Acid and Creatine Supplementation on Arsenic Methylation in a Randomized Controlled Trial of Bangladeshi Adults. *Eur. J. Nutr.* **2021,** *60* (4), 1921-1934.
9. Pfeiffer, C. M.; Huff, D. L. Gunter, E. W. Rapid and Accurate Hplc Assay for Plasma Total Homocysteine and Cysteine in a Clinical Laboratory Setting. *Clin. Chem.* **1999,** *45* (2), 290-2.
10. Hall, M. N.; Howe, C. G.; Liu, X.; Caudill, M. A.; Malysheva, O.; Ilievski, V.; Lomax-Luu, A. M.; Parvez, F.; Siddique, A. B.; Shahriar, H.; Uddin, M. N.; Islam, T.; Graziano, J. H. Gamble, M. V. Supplementation with Folic Acid, but Not Creatine, Increases Plasma Betaine, Decreases Plasma Dimethylglycine, and Prevents a Decrease in Plasma Choline in Arsenic-Exposed Bangladeshi Adults. *J. Nutr.* **2016,** *146* (5), 1062-7.
11. Yan, J.; Jiang, X.; West, A. A.; Perry, C. A.; Malysheva, O. V.; Devapatla, S.; Pressman, E.; Vermeylen, F.; Stabler, S. P.; Allen, R. H. Caudill, M. A. Maternal Choline Intake Modulates Maternal and Fetal Biomarkers of Choline Metabolism in Humans. *Am. J. Clin. Nutr.* **2012,** *95* (5), 1060-71.

12. Bobrowski-Khoury, N.; Sequeira, J. M.; Arning, E.; Bottiglieri, T. Quadros, E. V. Absorption and Tissue Distribution of Folate Forms in Rats: Indications for Specific Folate Form Supplementation During Pregnancy. *Nutrients* **2022,** *14* (12).
13. Rooney, M.; Bottiglieri, T.; Wasek-Patterson, B.; McMahon, A.; Hughes, C. F.; McCann, A.; Horigan, G.; Strain, J. J.; McNulty, H. Ward, M. Impact of the Mthfr C677t Polymorphism on One-Carbon Metabolites: Evidence from a Randomised Trial of Riboflavin Supplementation. *Biochimie* **2020,** *173*, 91-99.
14. Goodrich, J. A.; Wang, H.; Walker, D. I.; Lin, X.; Hu, X.; Alderete, T. L.; Chen, Z.; Valvi, D.; Baumert, B. O.; Rock, S.; Berhane, K.; Gilliland, F. D.; Goran, M. I.; Jones, D. P.; Conti, D. V. Chatzi, L. Postprandial Metabolite Profiles and Risk of Prediabetes in Young People: A Longitudinal Multicohort Study. *Diabetes Care* **2024,** *47* (1), 151-159.
15. Yu, T. Jones, D. P. Improving Peak Detection in High-Resolution Lc/Ms Metabolomics Data Using Preexisting Knowledge and Machine Learning Approach. *Bioinformatics* **2014,** *30* (20), 2941-8.
16. Yu, T.; Park, Y.; Johnson, J. M. Jones, D. P. Aplcms--Adaptive Processing of High-Resolution Lc/Ms Data. *Bioinformatics* **2009,** *25* (15), 1930-6.
17. Yu, T.; Park, Y.; Li, S. Jones, D. P. Hybrid Feature Detection and Information Accumulation Using High-Resolution Lc-Ms Metabolomics Data. *J. Proteome Res.* **2013,** *12* (3), 1419-27.
18. Uppal, K.; Soltow, Q. A.; Strobel, F. H.; Pittard, W. S.; Gernert, K. M.; Yu, T. Jones, D. P. Xmsanalyzer: Automated Pipeline for Improved Feature Detection and Downstream Analysis of Large-Scale, Non-Targeted Metabolomics Data. *BMC Bioinformatics* **2013,** *14*, 15.
19. Liu, Q.; Walker, D.; Uppal, K.; Liu, Z.; Ma, C.; Tran, V.; Li, S.; Jones, D. P. Yu, T. Addressing the Batch Effect Issue for Lc/Ms Metabolomics Data in Data Preprocessing. *Sci. Rep.* **2020,** *10* (1), 13856.
20. Schymanski, E. L.; Jeon, J.; Gulde, R.; Fenner, K.; Ruff, M.; Singer, H. P. Hollender, J. Identifying Small Molecules Via High Resolution Mass Spectrometry: Communicating Confidence. *Environ. Sci. Technol.* **2014,** *48* (4), 2097-8.
21. Uppal, K.; Walker, D. I. Jones, D. P. Xmsannotator: An R Package for Network-Based Annotation of High-Resolution Metabolomics Data. *Anal. Chem.* **2017,** *89* (2), 1063-1067.
22. Uppal, K.; Walker, D. I.; Liu, K.; Li, S.; Go, Y. M. Jones, D. P. Computational Metabolomics: A Framework for the Million Metabolome. *Chem. Res. Toxicol.* **2016,** *29* (12), 1956-1975.
23. Tamayo, L. I.; Kumarasinghe, Y.; Tong, L.; Balac, O.; Ahsan, H.; Gamble, M. Pierce, B. L. Inherited Genetic Effects on Arsenic Metabolism: A Comparison of Effects on Arsenic Species Measured in Urine and in Blood. Environ Epidemiol 2022, 6 (6), e230.
24. Pang, Z.; Lu, Y.; Zhou, G.; Hui, F.; Xu, L.; Viau, C.; Spigelman, A. F.; MacDonald, P. E.; Wishart, D. S.; Li, S. Xia, J. Metaboanalyst 6.0: Towards a Unified Platform for Metabolomics Data Processing, Analysis and Interpretation. *Nucleic Acids Res.* **2024**.
25. Pang, Z.; Chong, J.; Li, S. Xia, J. Metaboanalystr 3.0: Toward an Optimized Workflow for Global Metabolomics. *Metabolites* **2020,** *10* (5).
26. Darzi, Y.; Letunic, I.; Bork, P. Yamada, T. Ipath3.0: Interactive Pathways Explorer V3. *Nucleic Acids Res.* **2018,** *46* (W1), W510-W513.
